# Persistent, triple-virus co-infections in mosquito cells

**DOI:** 10.1186/1471-2180-10-14

**Published:** 2010-01-20

**Authors:** Nipaporn Kanthong, Nuanpan Khemnu, Sa-Nga Pattanakitsakul, Prida Malasit, Timothy W Flegel

**Affiliations:** 1 Department of Biotechnology, Faculty of Science and Technology, Rajamangala University of Technology Tawan-ok, Sriracha, Chonburi 20110, Thailand; 2 Division of Medical Molecular Biology, Office for Research and Development, Faculty of Medicine Siriraj Hospital, Mahidol University, Bangkok 10700, Thailand; 3 Medical Biotechnology Unit, National Center for Genetic Engineering and Biotechnology, National Science and Technology Development Agency, Science Park Pathumthani, 12120, Thailand; 4 Department of Biotechnology, Faculty of Science, Mahidol University, Rama 6 Road, Bangkok 10400, Thailand; 5 Centex Shrimp, Faculty of Science, Mahidol University, Rama 6 Road, Bangkok 10400, Thailand; 6 National Center for Genetic Engineering and Biotechnology (BIOTEC), National Science and Technology Development Agency, Klong 1, Klong Luang, Pratum Thani 12120, Thailand

## Abstract

**Background:**

It is known that insects and crustaceans can carry simultaneous, active infections of two or more viruses without showing signs of disease, but it was not clear whether co-infecting viruses occupied the same cells or different cells in common target tissues. Our previous work showed that successive challenge of mosquito cell cultures followed by serial, split-passage resulted in stabilized cultures with 100% of the cells co-infected with Dengue virus (DEN) and an insect parvovirus (densovirus) (DNV). By addition of Japanese encephalitis virus (JE), we tested our hypothesis that stable, persistent, triple-virus co-infections could be obtained by the same process.

**Results:**

Using immunocytochemistry by confocal microscopy, we found that JE super-challenge of cells dually infected with DEN and DNV resulted in stable cultures without signs of cytopathology, and with 99% of the cells producing antigens of the 3 viruses. Location of antigens for all 3 viruses in the triple co-infections was dominant in the cell nuclei. Except for DNV, this differed from the distribution in cells persistently infected with the individual viruses or co-infected with DNV and DEN. The dependence of viral antigen distribution on single infection or co-infection status suggested that host cells underwent an adaptive process to accommodate 2 or more viruses.

**Conclusions:**

Individual mosquito cells can accommodate at least 3 viruses simultaneously in an adaptive manner. The phenomenon provides an opportunity for genetic exchange between diverse viruses and it may have important medical and veterinary implications for arboviruses.

## Background

In a previous report [1], we described the successful establishment of stable, persistent co-infections of Dengue virus (DEN-2) and *Aedes albopictus *densovirus (*Aal*DNV) in a C6/36 mosquito cell line by sequential or simultaneous viral challenge followed by serial split-passage of whole cells. All of the cells in these cultures were co-infected and the two viruses were produced simultaneously without apparent negative effects on growth and morphology of the infected cells. The results revealed that insects infected with two viruses having common target tissues would have the potential to carry co-infected cells that could produce both viruses simultaneously. We hypothesized that repeating this process with a third virus could lead to the establishment of stable cell cultures with persistent, triple co-infections. In this brief communication, we describe the successful establishment of C6/36 mosquito cell cultures with triple co-infections of Japanese encephalitis virus (JE), Dengue virus (DEN-2) and *Aedes albopictus *densovirus (*Aal*DNV).

## Results and discussion

**Figure 1:**
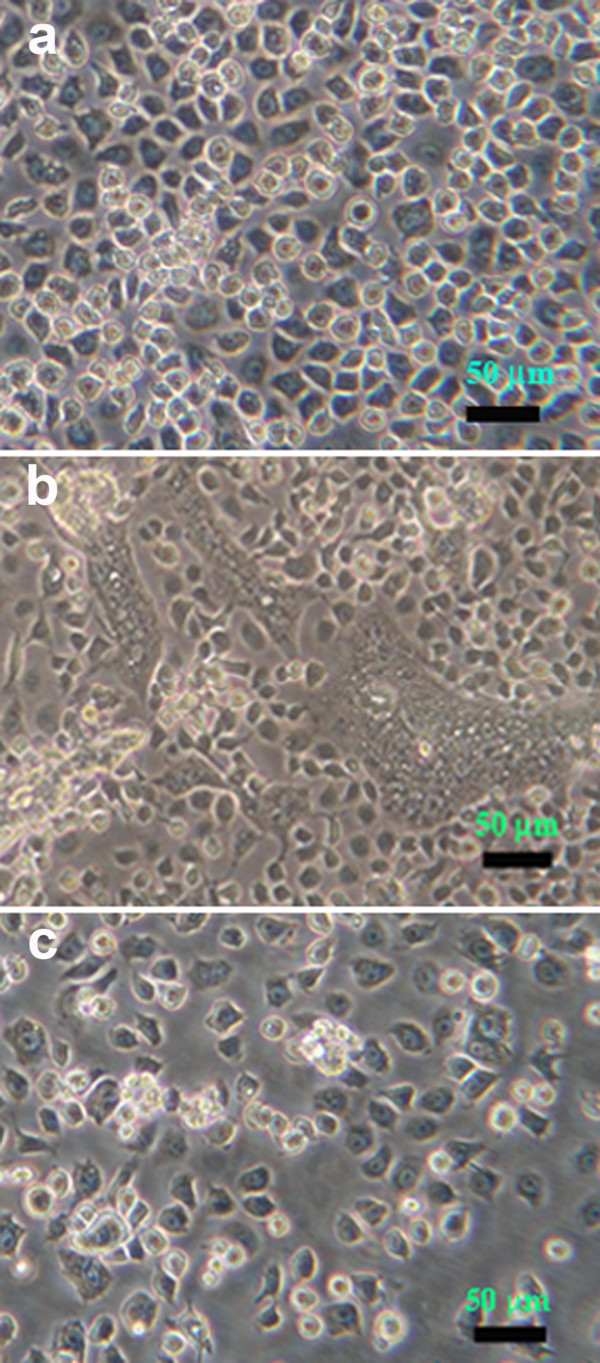
**Phase contrast photomicrographs of C6/36 cells**. (a) Naïve cells. (b) Cells with triple co-infections at passage 2 showing some cytopathology. (c) Cells with triple co-infections at passage 4 with morphology similar to that of naïve cells and of cells from higher passages.

When stable C6/36 cell cultures with dual, persistent infections of DEN-2 and *Aal*DNV were challenged with JE virus at MOI 0.1, the co-infected cultures showed a less severe response to JE than naïve C6/36 cells. The resulting super-challenged cultures were serially passaged at 5-day intervals. At early passages (1-4) in the split-passage process after JE challenge, some CPE was evident in the form of giant fusion cells (Figure 1b), but after the 5^th ^passage, very few giant cells could be found and the morphology of the culture cells resembled those in naïve cell cultures (Figure 1c), except that they tended to grow more slowly than the dually co-infected cells or naïve cells. These results were similar to those previously reported with DEN-2 super-challenge of cells persistently infected with *Aal*DNV, where CPE was less severe with the persistently infected cells than with acutely *Aal*DNV-infected cells or naïve cells challenged with DEN-2 [1,2].

**Figure 2:**
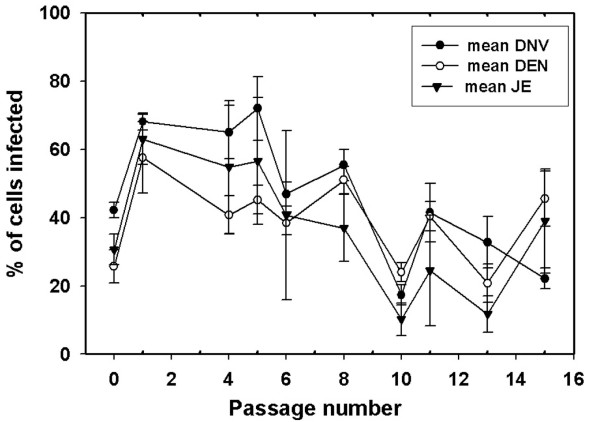
**Percentage of infected cells by flow cytometry**. Mean percent JE, DEN-2 and *Aal*DNV immunopositive cells detected by cell-flow cytometry during the course of serial split-passage after JE challenge of cells co-infected with DEN-2 and *Aal*DNV. Each data point represents the mean ± SD of 3 replicate cultures.

By flow cytometry, assay for the percentage of JE positive cells started out low (30 ± 4%) and increased within passage 1 to reach a mean value at 63 ± 7%. However, it dropped significantly (p < 0.05) thereafter. The mean value for passages 8-15 was 27 ± 6% (Figure 2). Similarly, the mean percentage of *Aal*DNV positive cells started low and then gradually increased with passage time to reach a mean value of 34 ± 4% from passages 8-15. The percentage of DEN-2 positive cells started out low (26 ± 5%) and increased within passage 1 to reach a mean value at 57 ± 10%. However, this dropped thereafter to give a mean value of 36 ± 4% for passages 8-15 (Figure 2). In summary, the general patterns for the three viruses were similar and the mean values for passages 8-15 were not significantly different (p = 0.351).

**Figure 3:**
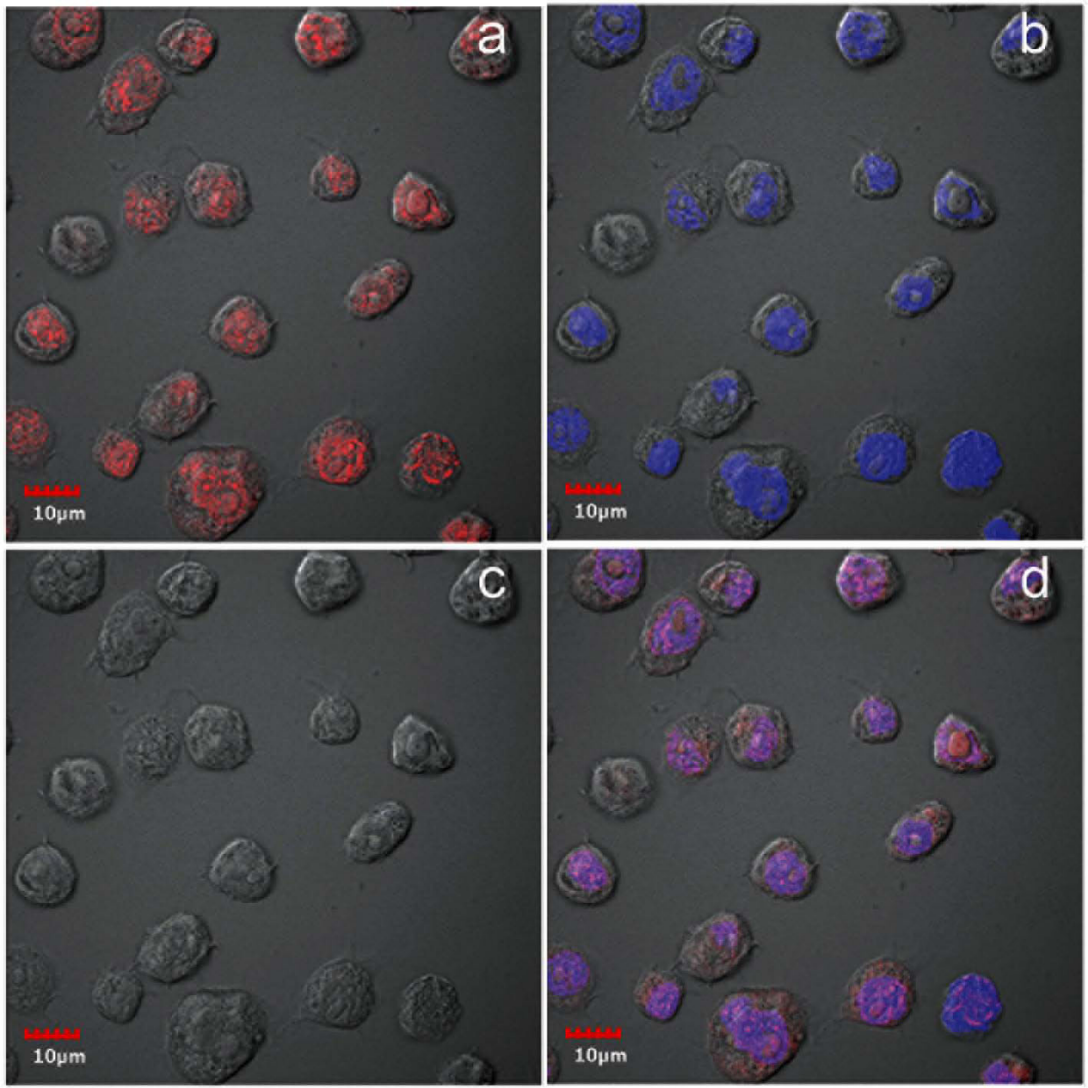
**Confocal microscopy of IFA for anti-JE**. Photomicrographs of immunofluorescence for anti-JE envelope protein in cells from cultures persistently co-infected with 3 viruses. Red = anti-JE and blue = pseudocolor for T0-PRO-3 iodide staining of DNA (nuclei). a = image for anti-JE only; b = image for T0-PRO-3 only; c = phase contrast image; d = combined images.

**Figure 4:**
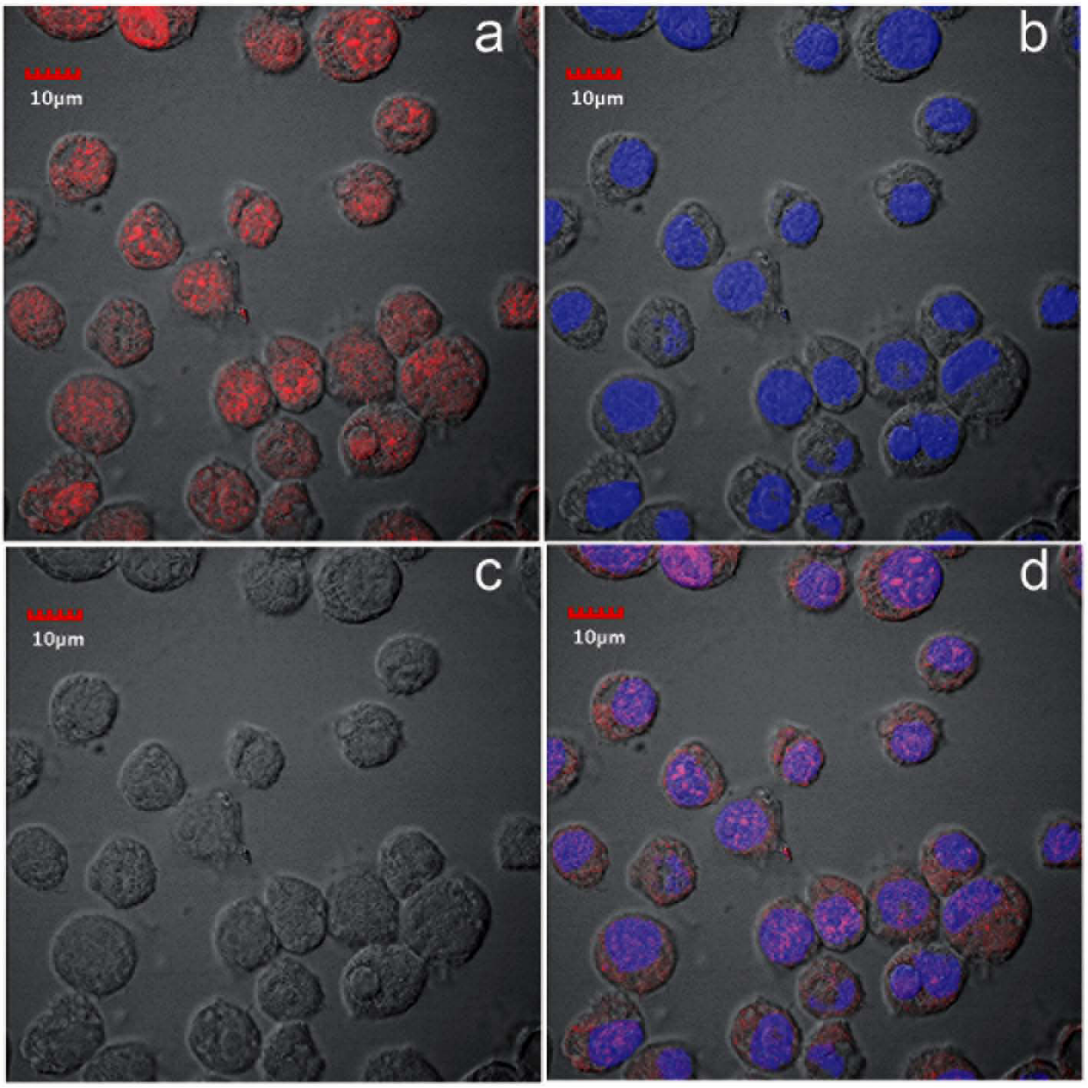
**Confocal microscopy of IFA for anti- *Aal*DNV**. Photomicrographs of immunofluorescence for anti-*Aal*DNV capsid protein in cells from cultures persistently co-infected with 3 viruses. Red = anti-*Aal*DNV and blue = pseudocolor for T0-PRO-3 iodide staining of DNA (nuclei). a = image for anti-*Aal*DNV only; b = image for T0-PRO-3 only; c = phase contrast image; d = combined images.

**Figure 5:**
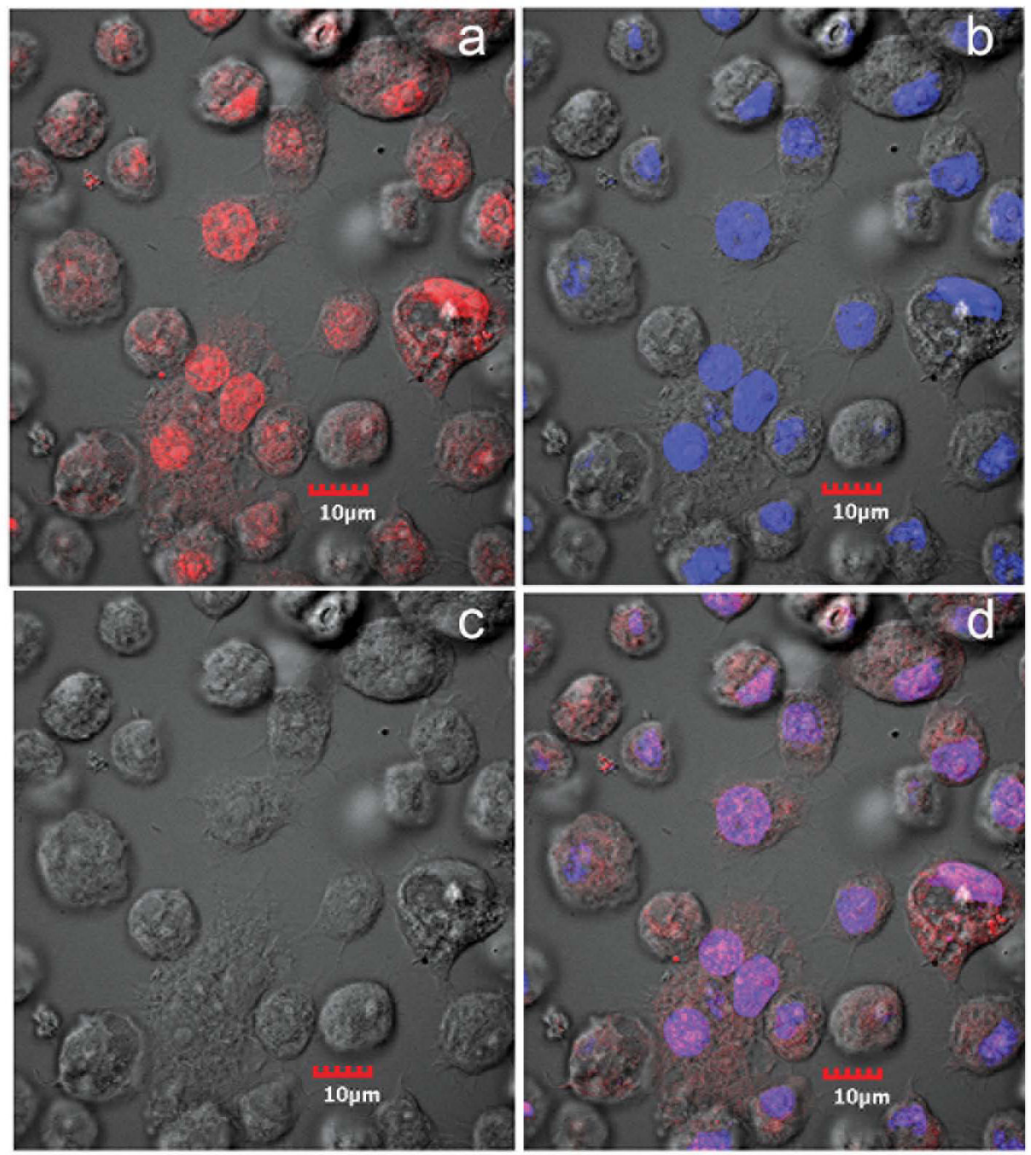
**Confocal microscopy of IFA for anti-DEN**. Photomicrographs of immunofluorescence for anti-DEN envelope protein in cells from cultures persistently co-infected with 3 viruses. Red = anti-DEN and blue = pseudocolor for T0-PRO-3 iodide staining of DNA (nuclei). a = image for anti-DEN only; b = image for T0-PRO-3 only; c = phase contrast image; d = combined images.

In contrast to flow-cell cytometry, immunofluorescence assay (IFA) by confocal microscopy revealed much higher numbers of positive cells. At passage 16 after challenge with JE, positive immunohistochemical reactions were seen exclusively in the nucleus (Figure 3) and the number of cells positive for JE at this passage was 99%. This contrasted with the mean value of 27 ± 6% for passages 8 to 15 that was obtained by flow cytometry. From the same passage-16 culture, IFA for *Aal*DNV capsid protein by confocal microscopy revealed positive immunofluorescence in both the nucleus and cytoplasm of infected cells, although the most intense signal was in the nucleus (Figure 4). The number of cells positive for *Aal*DNV at this passage was 100%, and again this contrasted with the value by flow cytometry (mean for passages 8-15 was only 34 ± 4%). As with the JE, positive IFA reactions for DEN-2 capsid protein by cells from the same passage 16 culture were seen exclusively in the nucleus (Figure 5) and 100% of the cells were immunopositive. Again, the mean percentage determined by flow cytometry for passages 8 to 15 was only 36 ± 4%. In summary, the proportions of immunopositive cells for the three viruses were 0.99, 1.0 and 1.0, indicating 99% (i.e., 0.99 × 1 × 1 × 100%) of the cells at this passage had triple co-infections. By the 16^th ^passage at a split ratio of 1/3, the originally challenged and washed insect cells would have been diluted by 3^16 ^= 4.3 × 10^7^. Assuming absence of any viral nucleic acid replication during cell division, no death of the originally challenged cells (unlikely) and no diminution in antigen during passage, only one in approximately 2 million cells would be expected to be immunopositive. Thus, the presence of 99-100% immunopositive cells for each of the 3 viral antigens indicated that there must have been replication of the viral nucleic acid responsible for antigen expression. This would not necessarily require production of viral particles, since viral nucleic acid could be transferred to daughter cells during cell division and with cells to culture flasks during split passage.

In an earlier report [1] stable, persistent infections of *Aal*DNV and DEN-2 alone in C6/36 cells were characterized by viral antigen located predominantly in the cytoplasm. By contrast, cells persistently co-infected with *Aal*DNV and DEN-2 [1] showed a shift in *Aal*DNV antigen from predominance in the cytoplasm to predominance in the nucleus, while DEN-2 remained exclusively in the cytoplasm. In a report on persistent infections by JE, also in C6/36 cells, it was reported [3] that viral antigen at early passage was predominant in the cytoplasm but that it was also present somewhat in the nucleus, while at late passage overall fluorescence was decreased and was distributed about equally in the cytoplasm and nucleus. This was similar to earlier results reported for cells persistently infected with DEN-2 alone [1].

In our triple co-infections, antigens for all 3 viruses were most strongly detected in the nucleus and only *Aal*DNV showed any signal in the cytoplasm. Thus, the distribution for *Aal*DNV antigen was the same as in previously described, dual co-infections (i.e., dominant in the nucleus but also present in the cytoplasm) while antigens for DEN-2 and JE were both found only in the nucleus. The curious intranuclear restriction for DEN-2 and JE was contrary to the expected cytoplasmic location for RNA viruses. Clearly, the addition of JE to the dual co-infection resulted in a shift of DEN-2 antigen from the cytoplasm to the nucleus and restriction of JE antigen to the nucleus in what could be interpreted as an adaptive, cellular response.

We have no explanation for the curious and unexpected distribution of JE and DEN-2 viral antigens exclusively in the nuclei of cells from the persistent, triple co-infections. Nor have we found any explanation for this phenomenon in the literature. There are only earlier reports describing cytoplasmic (dominant) and intranuclear (minor) fluorescence for viral antigens in C6/36 cells persistently infected with DEN-2 alone [1] or JE alone [3], without an explanation as to why. Reasons for shifts in location of viral antigens in C6/36 cells depending on single infection, dual co-infection or triple co-infection status are currently unknown, but the shifts clearly indicate that it involves some sort of cellular response to the presence of 2 or more viruses in simultaneous infections.

Another similarity between the previously reported persistent, single [3] or dual co-infections [1] and the current triple infections was the general decline in fluorescence (antigen quantity) for all of the viral antigens for high passage numbers. This was the cause of an apparent decline in percentage of infected cells by flow cytometry, despite 99% triple co-infection status revealed by confocal microscopy in our results and in a previous report [1]. As with viral antigen distribution, this indicates some kind of adaptive process that results in decreased expression of viral antigens with increasing passage number, until a stable state is reached.

Although DEN-2 and JEV antigens were detected in the nucleus, we expect that the viral RNA replicated in the cytoplasm and that antigens produced there were transported to the nucleus. In addition, the presence of antigens in the nucleus should not be equated with presence of viral particles there. Even though we did no electron microscopy with the triply co-infected cells, we do not expect that such examination would reveal the presence of recognizable viral particles, because of the low level of antigens present, and because recognizable viral particles were not seen in a previous study on dual co-infections of *Aal*DNV and DEN-2 [1]. On the other hand, that study did reveal that the co-infected cells produced infectious forms of both viruses in high amounts, and we expect (but did not test) that the triply infected cells would produce particles of all three viruses. However, even lack of infectious viral particles would not obviate the triple co-infection status of the cells.

## Conclusions

We have shown that stable, persistent, triple co-infections of viruses can be easily established without signs of disease in C6/36 mosquito cells by sequential viral challenge followed by serial split-passage of whole cells. This was achieved despite cytopathic effects that occurred at early passages after DEN-2 and JE super-challenge. Based on detection of viral antigens, serial addition of new viruses, starting with one in naïve cells, results in a trend for initially high levels of viral antigen followed by a gradual decline until stabilization from approximately 10-15 passages onward. Decreased fluorescence may lead to the erroneous conclusion from flow-cytometry results that the percentage of infected cells in the cultures is declining, even though the vast majority can be seen to be infected by confocal microscopy. The decline in viral antigens with stabilization, and the shifts in cellular location of the viruses with successive super-challenges indicate that the acquisition of new viruses and their stabilization in persistent, low-level infections is an adaptive process that occurs by currently unknown mechanisms. The results lend some support to the viral accommodation concept [4] concerning the capability of arthropods to carry one or more viruses in active, persistent infections without signs of disease. In addition, the revelation that two or more viruses can coexist in the same cells for long periods of time indicates that there may be an opportunity for genetic exchange, although the frequency of exchange would obviously depend on the degree of relatedness between the co-infecting viruses. This may have important medical and veterinary implications for arboviruses.

Altogether, the results suggest that existing or new insect cell cultures could easily carry undescribed viruses without showing gross and ultrastructural signs of disease or infection. Their presence could affect the results of experimental work with a different virus. For example, it has been shown here and in previous work [1,2] that existence of an underlying persistent infection with 1 or 2 viruses can reduce the cytopathic effect from a subsequent challenge with an additional virus. Thus, broad generalization about viral interactions based on results for viral challenge tests using insects and insect cells should be made with caution, especially when flow-cytometry is used to count numbers of infected cells. The same caution has been recommended for host-viral interaction studies in shrimp [5].

## Methods

### Manipulation of persistently-infected cell cultures

Cultures of C6/36 mosquito cells persistently co-infected with *Aal*DNV and DEN-2 were obtained from previous work [1]. Confluent cells from passage 30 in 25 cm^2 ^culture flasks (Costar, Corning) were split 1/3 and grown to confluence in 25 cm^2 ^culture flasks in 5 days in 5 ml Leibovitz's (L-15) medium containing 10% heat-inactivated fetal bovine serum (FBS), 10% tryptose phosphate broth (TPB) and 1.2% antibiotic (Penicillin G and Streptomycin). They were then challenged with Japanese encephalitis virus (JE) (Nakayama strain) at a multiplicity of infection (MOI) of 0.1. After incubation with the virus suspension for 2 hours with gentle shaking at room temperature, the medium was removed and fresh medium containing 2% FBS was added for further incubation (5 days) at 28°C. Then the supernatant medium was removed, the cells were suspended by knocking in 2 ml fresh L-15 medium containing 10% FBS before transfer to a new 25 cm^2 ^culture flask at 10^6 ^infected cells per flask followed by 5-days incubation. This process was repeated sequentially at 5-day intervals to establish persistently infected cultures. Mock-infected cells were run in parallel to the viral infected cells and served as negative controls. Tests were carried out in triplicate.

Japanese encephalitis virus (JE) (Nakayama strain) used in this work was obtained from the USArmed Forces Research Institute of Medical Sciences (AFRIMS) Bangkok through the courtesy of Ananda Nisalak and was stored in 20% fetal bovine serum at -80°C the Division of Medical Molecular Biology, Office of Research and Development, Faculty of Medicine Siriraj Hospital, Mahidol University, Bangkok. After thawing at room temperature, the stock was used as inoculum for monolayers of naïve C6/36 cells in Leibovitz's (L-15) medium containing 1% heat-inactivated fetal bovine serum (FBS), 10% tryptose phosphate broth (TPB) and 1.2% antibiotic (Penicillin G and Streptomycin). At day 4 after challenge, the supernatant solution was removed and used as inoculum for subsequent trials.

### Immunostaining for flow cytometry

Cultured insect cells were fixed with 4% paraformaldehyde in phosphate-buffer saline (PBS) for 20 minutes at room temperature, washed twice with PBS and treated with 0.1% triton X-100 in PBS. They were incubated with monoclonal antibody against the capsid protein of *Aal*DNV [1], 3H5 monoclonal antibody against DEN-2 envelope protein [6] and J93 monoclonal antibody against JE envelope protein. [antibodies were kindly provided by Ananda Nisalax at the USArmed Forces Research Institute of Medical Sciences (AFRIMS) Bangkok] at room temperature for 1 hour. They were washed again with 0.1% triton X-100 in PBS and incubated in a 50-fold dilution of anti-mouse IgG rabbit immune serum conjugated with FITC (F0261, DAKO) for 30 min at room temperature in the dark. After incubation, cells were washed once, resuspended in 1% formaldehyde in PBS and analyzed using a FACScan flow cytometer (Becton Dickinson). Mock cells were run in parallel and served as negative controls. At least 10,000 cells were gated by light scatter and collected in a list mode manner. Data analysis was performed using Cell Quest software (Becton Dickinson). The percentage of positive cells was determined from FITC fluorescence histograms using a region defined according to mock cells.

### Immunofluorescent staining for confocal microscopy

Cells from passage 16 were re-supended as described above and transferred for attachment to microscope slides. They were fixed with 4% paraformaldehyde in PBS for 15 min, washed twice with PBS, permeabilized with 0.1% Triton X-100 for 5 min and blocked with PBS containing 10% FBS. They were incubated for 1 hour with monoclonal antibody against the appropriate virus followed by incubation for 30 min with 1:500 dilution of fluorophore-labeled secondary antibody conjugate (Alexa Fluor 546 goat anti-mouse IgG, A-11001, from Molecular Probes) directed against the primary antibody. They were then washed with PBS before analysis. TO-PRO-3 iodide (T-3605, Molecular Probes) was used for nucleic acid counterstaining. Immunofluorescent-stained cells were analyzed by fluorescence microscopy and confocal laser microscopy (FV1000, Olympus). Two slides were prepared for each antibody assay. After scanning whole preparations to gain an overall impression, 6 representative fields were photographed (approximately 150 cells) in order to record the proportion of immunopositive cells.

## Authors' contributions

N Kanthong participated in the study design and the cell culture work, did the immunohistochemistry work, drafted the original manuscript and assisted in manuscript completion. N Khemnu participated in the cell culture work. SP and PM participated in the study design and interpretation of the results. TWF conceived the study, participated in the design and coordination and took major responsibility for writing the manuscript. All authors read and approved the final manuscript.
